# Upregulation of Myocardial and Vascular Phosphodiesterase 9A in A Model of Atherosclerotic Cardiovascular Disease

**DOI:** 10.3390/ijms19102882

**Published:** 2018-09-22

**Authors:** Daniel Priksz, Mariann Bombicz, Balazs Varga, Andrea Kurucz, Rudolf Gesztelyi, Jozsef Balla, Attila Toth, Zoltan Papp, Zoltan Szilvassy, Bela Juhasz

**Affiliations:** 1 Department of Pharmacology and Pharmacotherapy, Faculty of Medicine, University of Debrecen, H-4032 Debrecen, Hungary; priksz.daniel@pharm.unideb.hu (D.P.); bombicz.mariann@pharm.unideb.hu (M.B.); varga.balazs@pharm.unideb.hu (B.V.); kurucz.andrea@pharm.unideb.hu (A.K.); gesztelyi.rudolf@pharm.unideb.hu (R.G.); szilvassy.zoltan@med.unideb.hu (Z.S.); 2 Institute of Internal Medicine, Faculty of Medicine, University of Debrecen, H-4032 Debrecen, Hungary; balla.jozsef@med.unideb.hu; 3 Division of Clinical Physiology, Faculty of Medicine, University of Debrecen, H-4032 Debrecen, Hungary; atitoth@med.unideb.hu (A.T.); pappz@med.unideb.hu (Z.P.)

**Keywords:** echocardiography, endothelial dysfunction, cardiac dysfunction, phosphodiesterase 9A, atherosclerosis, rabbit model

## Abstract

Atherosclerosis is strongly associated with cardiac dysfunction and heart failure. Besides microvascular dysfunction and diminishment of the cardiac nitric oxide-Protein Kinase G (NO-PKG) pathway, recent evidence suggests that phosphodiesterase 9A (PDE9A) enzyme has an unfavorable role in pathological changes. Here, we characterized a rabbit model that shows cardiac dysfunction as a result of an atherogenic diet, and examined the myocardial PDE9A signaling. Rabbits were divided into Control (normal diet) and HC (atherogenic diet) groups. Cardiac function was evaluated by echocardiography. Vascular function was assessed, along with serum biomarkers. Histological stains were conducted, expression of selected proteins and cyclic guanosine monophosphate (cGMP) levels were determined. Signs of diastolic dysfunction were shown in HC animals, along with concentric hypertrophy and interstitial fibrosis. Endothelial function was diminished in HC rabbits, along with marked reduction in the aortic lumen, and increased left ventricle outflow tract (LVOT) pressures. A significant increase was shown in myocardial PDE9A levels in HC animals with unchanged vasodilator-stimulated phosphoprotein (VASP) phosphorylation and cGMP levels. Upregulation of PDE9A may be associated with early stage of cardiac dysfunction in atherosclerotic conditions. Since PDE9A is involved in cGMP degradation and in deactivation of the cardioprotective PKG signaling pathway, it may become an encouraging target for future investigations in atherosclerotic diseases.

## 1. Introduction

Dyslipidemia is a well-documented risk factor for cardiovascular disorders and many different preclinical and clinical trials proved that the elevation of low-density lipoprotein (LDL) serum level causes atherosclerotic cardiovascular disease [1]. Atherosclerosis is associated with ischemic injury and myocardial infarction, and it was also shown that dyslipidemia is also an independent risk factor for heart failure (HF) [2,3].

The nitric-oxide (NO)-cyclic guanosine monophosphate (cGMP)-Protein Kinase G (PKG) pathway is a substantial cascade that regulates cardiac function, with downstream targets being responsible for cardioprotective signaling, and regulation of myocardial relaxation via the giant elastic protein, titin [4,5,6]. Subsequently, the downregulation of this pathway may be greatly responsible for diastolic dysfunction (DD), which is the precursor of diastolic heart failure (HF with preserved ejection fraction, HFpEF) and characteristic in systolic heart failure as well (HF with reduced ejection fraction, HFrEF) [7,8]. Atherosclerosis-associated endothelial inflammation in coronary microvasculature, with increased oxidative stress, causes diminished NO bioavailability and disrupted PKG signaling, which results in titin malfunction and increased myocyte stiffness, as well as inflammation-associated myofibroblast formation with interstitial fibrosis [4]. The key messenger cGMP could not only be generated by soluble guanyl cyclase (sGC), triggered by NO, but also by guanyl cyclase/natriuretic peptide receptor-A and B (pGC), when natriuretic peptides (NPs) are present [9]. Diminished B-type natriuretic peptide (BNP) expression may be a consequence of multiple factors (obesity, insulin resistance, left ventricle (LV) wall stress), causing a decline in myocardial cGMP concentration [10,11,12]. Decreased levels of cGMP may also result from increased expression and activity of phosphodiesterase enzymes (PDEs), that metabolize cyclic AMP (cAMP) and cGMP. A recent study revealed that phosphodiesterase type 9A (PDE9A), being highly selective for cGMP, is responsible for the degradation of NP-derived cGMP pools only [13]. Decreased levels of natriuretic peptides (NPs) or degradation of NP-derived cGMP by PDE9A contributes to the pathology of HFpEF, as it was shown on PDE9A global knockout mice that underwent a pressure-overload technique [13]. Altering this pathway could lead to a new direction in drug discovery—i.e., by pharmacological augmentation of the NP-derived cGMP system, besides the well-known PDE5 inhibitors. In recent years, this theory was further evidenced by the fact that enhancement of NP signaling by inhibiting neprilysin, (the enzyme that metabolizes natriuretic peptides), have had promising results in clinical trials (PARAGON-HF trial) [14].

In investigating disease pathology, rabbit models represent a tool that shares advantages with both small and large animal models: could be maintained on relatively low costs, but having a heart physiology (including Ca-handling, ion channels and actin-myosin structure) similar to human [15,16,17,18,19]. Moreover, as being extremely sensitive to atherogenic diets and having comparable lipoprotein homeostasis to human, rabbits models could be successfully used in fields of cardiovascular research [20].

The major objective of the present study was to evaluate whether cardiac dysfunction caused by atherogenic diet affects myocardial PDE9A expression and cGMP levels. As a result of a high-cholesterol diet, the rabbit model characterized here shows signs of diastolic and mild systolic dysfunction, with the presence of interstitial fibrosis, atherosclerosis and endothelial dysfunction, evidence revisited with an integrative methodological approach. Parallel molecular biological examinations showed increased expression of cardiac PDE9A, but unchanged cGMP levels and vasodilator-stimulated phosphoprotein (VASP) phosphorylation, referring to unaltered PKG activity. As upregulated PDE9A limits the elevation of cGMP levels and blocks cardioprotective PKG signaling, it may be a favorable drug target in atherosclerosis-associated cardiac dysfunction.

## 2. Results

### 2.1. Atherogenic Diet Induces Cardiac Dysfunction

Significant differences were found in the echocardiographic parameters of the HC group compared to the Control, especially in parameters referring to diastolic function (Table 1 and Table 2). In the HC group, the area of the left atrium, left ventricle mass, relative wall thickness, left ventricle outflow tract velocities and pressures showed an increase, whereas E/A ratio, septal e’/a’ were lower, with a lengthening in deceleration time, as well as elevated E/e’ ratios. Isovolumic relaxation time was dramatically extended, with an elevated myocardial performance index. Regarding systolic function, the fractional shortening (FS) and the ejection fraction (EF) of left ventricle were slightly decreased, but remained in the normal range, while mitral annular plane systolic excursion (MAPSE) values were unaffected by the treatment. Speckle tracking data showed decreased global longitudinal strain (GLS) values in HC animals compared to Controls.

### 2.2. Increased Left Ventricle Mass and Bodyweight in HC Animals

Animals in the HC group gained significant weight, and left ventricle mass was significantly increased compared to the Controls (Table 3). Since bodyweights fluctuated, LV mass was normalized to tibial length, as a more standard parameter. Lung and kidney wet to dry tissue ratios did not differ significantly among groups.

### 2.3. Atherogenic Diet Elevates Serum Lipid Parameters

Values of serum lipid parameters and biomarkers are shown in Table 4. All parameters of Control animals remained in the physiologic range, in contrast, serum cholesterol, low-density lipoprotein (LDL), and apolipoprotein B (ApoB) values of HC animals were dramatically increased compared to Controls. LDL and ApoB, as well as high-density lipoprotein (HDL) and apolipoprotein A (ApoA) levels were well-correlated. Atherogenic index and ApoB to ApoA ratio increased significantly. No changes were observed in hepatic enzyme levels. Troponin T and creatinine were elevated in HC animals, but creatine kinase MB isoenzyme (CK-MB) was not. As a limitation, serum N-terminal pro-B-type natriuretic peptide (NT-pro-BNP) levels of all animals were below the detectable limit, thus statistical analyses could not be accomplished.

### 2.4. Atherogenic Diet and Endothelial Dysfunction with Increased Aortic PDE9A Expression

**Figure 1:**
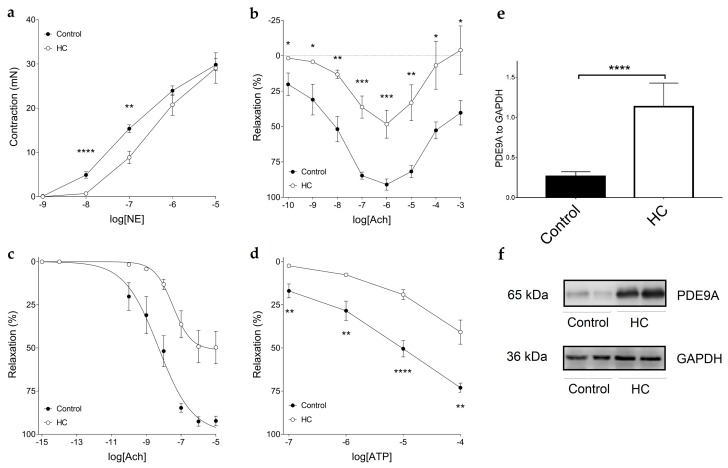
The effect of norepinephrine (NE), acetylcholine (Ach) and adenosine 5′-triphosphate ATP on the thoracic aorta isolated from rabbits treated with normal (Control group) and atherogenic (HC group) chow (**a**). Axis *x* shows the common logarithm of molar concentration of NE, while axis y indicates the contractile force of the aortic rings (over the resting level). **(b**) denotes the effect of acetylcholine (Ach), while the panel (**c**) displays separately the Ach-evoked (so-called endothelium-dependent) relaxation in the same model. On panel (**b**), the symbols represent the effect of Ach averaged within the groups (± standard error of the mean, as SEM), and asterisks denote the significance level of differences between the groups. Axis *x* shows the common logarithm of molar concentration of Ach, while axis *y* indicates the effect as a percentage decrease in the initial contractile force of aortic rings. Symbols on panel (**c**) show the modified effect values averaged within the groups (±SEM), and the lines denote the Hill equation fitted to the averaged modified Ach E/c data. All aortic rings underwent a pre-contraction evoked by norepinephrine (NE) before the administration of Ach. (**d**) shows the effect of adenosine 5′-triphosphate (ATP) on the thoracic aorta isolated from groups. All aortic rings underwent a pre-contraction evoked by norepinephrine (NE). Axis *x* shows the common logarithm of molar concentration of ATP, and axis *y* indicates the effect as a percentage decrease in the initial contractile force of aortic rings. The symbols represent the effect of ATP averaged within the groups (±SEM). Panel (**e**) shows the expression of PDE9A in thoracic aorta of rabbits, by using Western blot technique. (**f**) shows representative blots of anti-PDE9A and anti-GAPDH incubated membranes. Asterisks denote the level of statistical significance concerning differences between the two groups (* *p* < 0.05; ** *p* < 0.01; *** *p* < 0.001; **** *p* < 0.0001).

The contractile forces evoked by norepinephrine (NE) were significantly different between the Control and HC groups at 10 and 100 nmol/L NE concentrations, as a weakened response to NE was observed in the HC group (Figure 1a). In both the Control and HC groups, acetylcholine (Ach), up to 1 µmol/L concentration, evoked relaxation, while higher Ach concentrations produced contraction. However, atherogenic diet dramatically decreased the relaxation of aortic rings evoked by Ach with the preservation of the Ach-induced contraction (measured from the maximally relaxed state) (Figure 1b). The *E*_max_ and logEC_50_ values yielded by the Hill equation (fitted to the individual modified Ach E/c data) significantly differed between the Control and HC groups: *E*_max_ showed a considerable decrease, whereas logEC_50_ increased moderately in response to the atherogenic diet (Figure 1c and Table 5). ATP caused a relaxation in the aortic rings pre-contracted with NE. This effect was, however, significantly stronger in the Control group, thus the atherogenic diet also impaired the ATP-induced arterial relaxation (Figure 1d). This finding corroborates the results obtained with the use of Ach, the classic vasodilator molecule. Western blots were carried out on stored thoracic aorta samples. Figure 1e,f show the increased expression of PDE9A in aortic samples of HC animals in comparison to Controls.

### 2.5. Plaque Coverage and Interstitial Fibrosis in HC Animals

**Figure 2:**
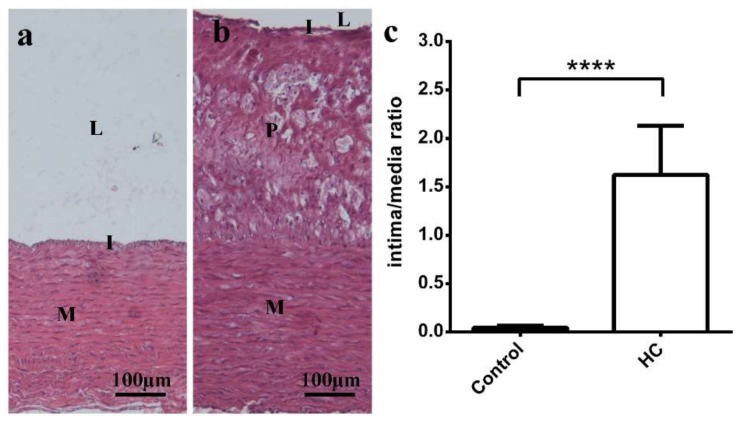
Histological analysis of aortic sections. (**a**) shows a healthy control sample with intact endothelium. Foamy atherosclerotic plaque observed in HC animals is shown on panel (**b**). Graph (**c**) demonstrates intima/media ratio, which was significantly increased in HC group compared to Control (**** *p* < 0.0001). M: vascular media; I: intimal layer, L: lumen; P: atherosclerotic plaque.

**Figure 3:**
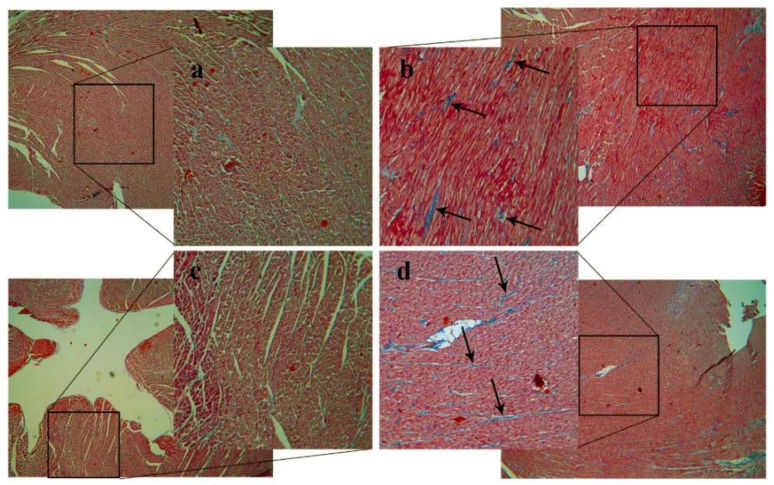
Masson’s trichrome stains of the left ventricle. Interstitial fibrosis was examined in different regions of the myocardial tissue. (**a**,**b**) are from the midline of the ventricle, (**c**,**d**) from the apical region. HC samples (**b**,**d**) show blue fibrotic lines (arrows), while Controls (**a**,**c**) do not. 40× (outer pictures) and 100× (inner pictures) magnification.

The intimal layer of the HC rabbits was significantly thicker compared to the controls (Figure 2a,b). Due to the thick foamy plaque layer, intimal thickness of HC animals turned out to be more than 1.5-times the size of the vascular media. Differences between the groups are also demonstrated by the calculated intima/media ratios: 1.623 ± 0.079 vs. 0.043 ± 0.004, HC vs. Control, respectively (Figure 2c). Samples of HC heart tissue showed mild fibrosis (blue lines, Masson’s trichrome stain), as demonstrated in Figure 3b,d, that was not observable in Control hearts (Figure 3a,c).

### 2.6. Atherogenic Diet Correlates with the Upregulation of Myocardial PDE9A and PKG1

**Figure 4:**
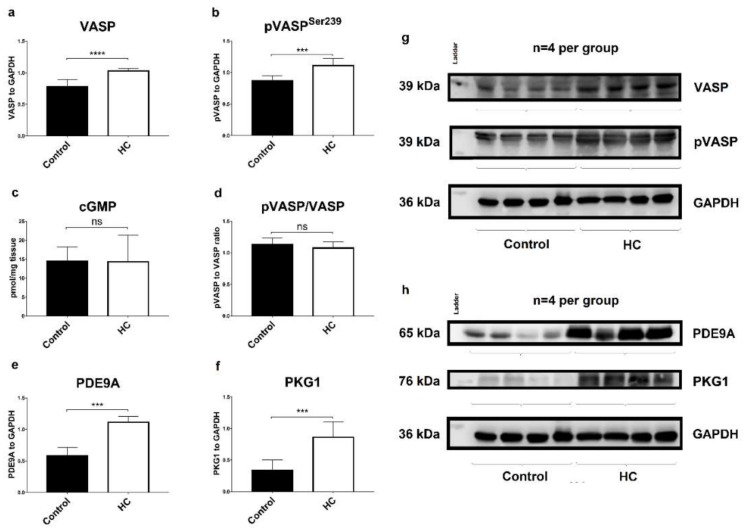
Outcomes of Western blot analyses and cGMP assays. Average band size for PDE9a was observed at around 65 kDa (*n* = 4 per group). The expression of PDE9A was significantly higher in LV samples of HC animals in comparison to Controls (*** *p* < 0.001) (**e**). No significant differences were found in myocardial cGMP levels (expressed as pmol/mg of tissue, ELISA) of endpoint groups (**c**). Although both VASP and pVASP expression was significantly increased in HC group (*** *p* < 0.001 and **** *p* < 0.0001, respectively) (**a**,**b**), pVASP to VASP ratio was unchanged (**d**) and was well-correlated with cGMP levels. Expression of PKG1 enzyme was also elevated in HC group (**f**). Panels (**g**,**h**) show representative blots. Student’s *t*-test was used to estimate differences between the samples of HC and Control animals.

Results of Western blot and cGMP analyses are presented in Figure 4. Protein was isolated from left ventricle samples, and results are shown as an average of 3 repeated electrophoreses, with representative blots. When standardized to GAPDH, a two-fold increase was observed in PDE9A levels in the LV samples of HC animals, compared to Controls (*** *p* < 0.001, Figure 4e). Expression of VASP (Figure 4a) and pVASP^Ser239^ (Figure 4b) increased significantly in HC group, with unchanged pVASP/VASP ratios (Figure 4d), referring to PKG1 activity. Accordingly, cardiac cGMP levels did not differ significantly among groups (Figure 4c).

## 3. Discussion

### 3.1. Cardiac Dysfunction

As a precursor of heart failure (HF), diastolic dysfunction (DD) can be present even before clinical symptoms of HF appear, and despite its clear clinical significance, DD is an entity which remains poorly understood. Here, we present a rabbit model that shows signs of DD in a similar way to human patients. Echocardiographic outcomes of HC rabbits showed decreased E/A ratios, lengthened deceleration time (DecT), and increased E/e’ which are reflective of increased filling pressures, according to current recommendations [21]. Diastolic dysfunction was further evidenced by an increase in the left atrial (LA) area, lengthened isovolumic relaxation and increased Tei-index. Left ventricle outflow tract (LVOT) pressures and velocities were also increased after treatment, which also confirms pathology. At the same time, right ventricle parameters were unaffected: showed no signs of congestion or pulmonary edema, referring to the fact that diastolic dysfunction was in a preclinical stage.

An increase in relative wall thickness (RWT) and LV mass with normal internal diameter of chambers was observed in HC animals, referring to concentric hypertrophy, which further confirms pressure overload and diastolic pathology. In accordance, after using Masson’s trichrome stain, interstitial fibrosis was detected in HC animals (Figure 3). Several studies demonstrated that LV hypertrophy may be partly responsible for HFpEF phenotype, with underlying mechanisms including vascular dysfunction, extracellular matrix abnormalities (fibrosis) and altered myocyte stiffness [22]. Fibrosis is characteristic in HFpEF, and may be a result of microvascular endothelial dysfunction and macrophage infiltration [23]. Furthermore, despite the normal LV ejection fraction [24], HC animals showed significantly decreased GLS values, as an early sign of deteriorated global LV systolic function, in strong accordance with findings described among human patients [25]. Our findings also support observations by others, namely that high blood cholesterol is strongly associated with the worsening of longitudinal and circumferential strain parameters in rabbits. Under similar conditions, decline in systolic and diastolic function was also shown on isolated myocytes, in a condition referred to as “cholesterol cardiomyopathy” even though these results were not confirmed by echocardiography. Although strain rate imaging has not been integrated into the diagnostic criteria yet, it seems to be a promising novel method for future classification of various cardiac pathologies.

### 3.2. Dyslipidemia and Endothelial Dysfunction

Atherogenic index, LDL and ApoB concentrations increased (Table 4), and atherosclerotic lesions accompanied by elevated intima/media ratios were found along with elevated intima/media ratios in Californian-New Zealand (CAL-NZW) rabbits on a high-cholesterol and fat diet (Figure 2). Although coronary vessels were not examined, we presume that coronary atherosclerosis may also be present as strong clinical evidence suggests that the presence of abdominal aortic plaques is highly associated with coronary artery disease (CAD) [26]. Nevertheless, it is assumed that significant thrombotic events and acute myocardial infarction (AMI) were absent, as AMI serum markers (CK-MB) were not elevated. Even though a marked increase (around three times of the Control value) was observed in troponin-T serum levels, values remained below the limit suggestive of AMI (where it is elevated 50–100 times of the normal, or above 100 ng/L). Moreover, elevation of troponin T is strongly associated with the extent of LV relaxation abnormalities (especially with decreased Tissue Doppler peak e’ wave velocity) in HFpEF patients, as it had been shown previously [27]. These observations were further confirmed by our results, as HC animals showed elevated troponin T levels along with lower peak e’ wave velocities (Table 2 and Table 4). Naturally, on the basis of measurements of serum parameters alone, we cannot exclude the possibility that there may have been previous thrombotic events. Nevertheless, our assumptions were further corroborated by the results of histological stains where we failed to observe any visible sign of previous infarction or necrosis.

Hypercholesterolemia predisposes patients to atherosclerosis, and plaque formation causes impaired endothelium-dependent vasorelaxation, even in early stages of the disease [28]. Impaired vasorelaxation is the hallmark of endothelial dysfunction, with the pivotal role of NO, the primary vasodilator mediator released by the endothelium and—according to a newly introduced view—by arterial smooth muscle [29]. Coronary artery disease and microvascular inflammation are thought to be major contributors to HFpEF, over increased afterload, according to a novel paradigm published lately [30,31]. This theory highlights the role of inflammation in vasculature, causing reduced NO bioavailability, low cGMP levels and PKG activity in neighboring myocytes. Subsequently, diminished NO bioavailability is a key factor of endothelial dysfunction and atherosclerosis, and may contribute to disrupted PKG signaling in myocytes, which increases resting tension, stiffness, fibrosis, and diastolic failure. Here, endothelial function was assessed by using Ach, and it showed that the atherogenic diet dramatically decreased the relaxation of vessels evoked by Ach, but the Ach-induced contraction was preserved. Beside the “classical” vasodilator substance Ach, endothelial dysfunction was also verified with the help of ATP, and similar outcomes were achieved. Interestingly, expression of PDE9A was found to be increased in aortic samples of HC animals, which may further contribute to decreasing vascular cGMP levels and impaired vasorelaxation.

Additionally, marked circumferential plaque coverage was observed in aortic root samples of HC animals, with significant narrowing of the vessel lumen (around 50%), in accordance with data shown by others [20]. Therefore, we can conclude that together with impaired relaxation and increased aortic stiffness, this constriction further contributes to diastolic dysfunction in a mechanical way, as reduced aortic diameter results in increased pressure. It generates pressure overload, that manifests in increased afterload, which in turn induces structural changes in myocardium, in a similar way to the classical transverse aortic constriction (TAC) models of heart failure [32,33,34]. Increased pressures were confirmed by echocardiographic measurements, showing significantly elevated LVOT maximal and mean pressure gradients and flow velocities (Table 1).

Out of the biomarkers of the disease, it is only NT-pro-BNP (inactive form of BNP) that is routinely used for diagnosis, although there are several other markers of inflammation; oxidative stress and endothelial dysfunction (interleukin-6 (IL-6), tumor necrosis factor α (TNF-α), pentraxin-e, vascular cell adhesion molecule 1 (VCAM-1), Soluble ST2, *E*-selectin) suggesting the progression of pathology [4]. What brought limitation to this report was that we were unable to measure pro-BNP levels as they were below the detection limit. As a result, we could not determine if DD tends to be translated into HF in these animals.

### 3.3. Upregulation of Cardiac PDE9A in Correlation with Cardiac Dysfunction

A primary outcome of this study is that PDE9A upregulation was shown in LV samples of HC animals for the first time—to our knowledge—in atherosclerotic conditions. Upregulation of PDE9A in cardiac (and cognitive) diseases is of great interest, since it was first shown that PDE9 regulates NP-derived cGMP pools, and thus it may even be a more potent contributor to heart failure pathology than PDE5 [13]. PDEs metabolize cyclic nucleotides (cAMP and cGMP) by hydrolyzing them to 5′-monophosphates, and the two isoforms PDE5 and PDE9 expressed in the heart are highly selective to cGMP. The original assumption, that PDE5 inhibitors are effective in the treatment of heart failure was not fully proven, and attempts failed to show substantial benefit in human patients, despite the encouraging data obtained from animal experiments [35]. Moreover, elevation of PDE5 in HFpEF was not confirmed, and clinical trials with PDE5-inhibitors in HFpEF produced neutral results [36,37,38]. These outcomes led to the question whether there was another way to control cGMP levels. Research finally resulted in the discovery of PDE9 and its role in heart failure. PDE9 has the highest selectivity for cGMP, and is expressed in brain, kidney and heart tissues. Studies revealed that the expression of PDE9A is elevated in mice and human myocardium, both in HFpEF and HFrEF [13]. Furthermore, inhibition or genetic deletion of the enzyme showed promising results in experimental heart disease (TAC model), by alleviating symptoms, restoring systolic function and inhibiting fibrosis as it has been published recently [13]. While PDE5 is localized at the Z-disc, PDE9A can be found at the T-tubular membrane. This compartmentalization may be responsible for the difference in their effects: although the two isoforms share pathways, they also have distinct downstream effectors. Therefore, this phenomenon may clarify how PDE9A is only responsible for the degradation of NP-derived cGMP pools [13,35].

Here, in connection with the PDE9A, VASP and p-VASP^Ser239^ protein expressions were also measured. Firstly, we experienced a significant increase in unphosphorylated VASP expression level in the myocardial tissues of atherosclerotic animals, which may corroborate with the results of other investigators, however, to our knowledge, this is the first study that reported increased myocardial VASP expression in atherosclerotic CVD. Sartoretto et al. showed increased VASP expression after TAC procedure and in hypertrophic conditions, and this may further strengthen the hypothesis that atherosclerosis, endothelial dysfunction and vessel wall stiffness may generate pressure overload similarly to the TAC model, consistent with our echo results (increased LVOT pressures, wall thickness, E/e’ ratios) presented here [39]. It was also shown that VASP family appear to modulate diverse cellular responses as migration and cell–cell junctions, as TAC procedure increases VASP expression and results in hyperthrophic remodeling, while cardiac fibrosis and hypertrophy markers are elevated in VASP^−/−^ knockout mice. It seems that VASP regulation is a complex process, and both hypo- or hyperctivation leads to pathological changes in the myocardium [40].

More importantly, activity of PKG1 enzyme may be indirectly measured by p-VASP^Ser239^/VASP ratios, as PKG uniquely phosphorylates VASP protein at Ser239 [41]. Elevated cGMP levels and increased activity of the PKG enzyme is considered to initiate cardioprotective signaling, which was shown in different pathological conditions in several studies. Firstly, this PKG is one of the downstream effectors for natriuretic peptides, released by myocytes after increased pressure load. Besides natriuretic and vasodilating effects, NPs transfer antifibrotic and antihypertrophic signals via the cGMP/PKG system [42]. The cardioprotective actions of cGMP/PKG pathway was also shown in I/R injury. Activation of PKG is essential for protective effects of ischemic post-conditioning [43]. Moreover, our research team previously showed that preconditioning protects against I/R injury, and this favorable effect depends on increased myocardial cGMP levels [44]. Another indirect evidence for the protective actions of cGMP/PKG system, is that inhibition of PDE5A may be beneficial against heart failure in some clinical setting. However, data about the cardioprotective effects of PDE5 inhibitors is a subject of debate, as the clinical trials failed to show clear benefit, in contrast to encouraging results obtained from animal experiments [45]. In summary, in our experimental setting, it is possible that the upregulation of PDE9A limits the initiation of the cardioprotective cGMP/PKG pathway, which was evidenced by direct measurement of myocardial cGMP content and also by measuring PKG kinase activity by VASP Ser239 phosphorylation.

Our data shows cardiac PDE9A upregulation in atherosclerotic conditions, which may be a consequence of DD. As far as intracellular cGMP is concerned, it may confirm earlier works regarding the role of PDEs in the regulation of cardiac cyclic nucleotide cross-talk signaling network. The unchanged level of cGMP could be explained by the adaptation of PDE5A to PDE9A upregulation, as when one PDE is upregulated, the activity of another may compensate for it [13]. As both compartmentalization of PDE enzymes and augmentation of the cGMP system in cardiac pathology are of intense interest, several unresolved questions will presumably be addressed in further investigations.

### 3.4. Translational Aspects and Future Directions

In the light of translational medicine, it is concluded that even though it is a well-established fact that hyperlipidemia is a risk factor for cardiovascular diseases, obviously, dietary cholesterol intake does not result in the same rapid and serious pathophysiological changes in humans as in rabbits. However, it can be assumed that high-fat diets, obesity and CAD contribute to diastolic dysfunction in human patients as well. Accordingly, the rabbit model presented here may offer a good approach in mimicking such conditions. Rabbits fed with high-cholesterol diet a for long time (e.g., 40 weeks) or with a diet of higher cholesterol content (2%) develop serious CAD, AMI and systolic HF [46]. However, under moderate conditions and within a shorter time span, early-stage atherosclerosis develops with endothelial damage, diastolic dysfunction and deteriorated systolic strain parameters.

Rabbits may serve as good models in many fields for cardiovascular research as their cholesterol homeostasis and the physiology of their heart resembles that of humans. Admittedly, in specific fields, canine or sheep models may be superior due to the fact that rabbits have a much lower heart rate reserve, which affects experiments aimed to investigate the effect of exercise training [17]. As for small rodents (mice and rats)—especially transgenic ones—they are extremely useful in various fields of cardiovascular research, but not in modeling atherosclerosis. Rats and mice are highly resistant to atherogenic diets, moreover, they have high HDL levels but lack CETP (cholesteryl ester transfer protein), unlike humans [17]. Accordingly, it is rabbit models that are still the most popular for investigations related to atherosclerosis, CAD and comorbidities.

Here, we showed that cardiac PDE9A is upregulated even in an early stage of atherosclerosis-induced cardiac dysfunction. If this pathway is essential in the human body, identifying missing messengers in the natriuretic peptide-cGMP signaling pathways, investigating crosstalk between PDEs or clarifying the role of downstream effectors (PKG, titin) may bring possible treatment strategies to human medicine (i.e., PDE9 inhibitors, Ca-sensitizers, soluble guanyl cyclase (sGC) activators, neprilysin inhibitors). Moreover, augmentation of NO-cGMP pathway could lead to new directions with more specific approaches, which have been recently highlighted in the guideline of European Society of Cardiology, with the implementation of angiotensin receptor-neprilysin inhibitors (ARNI) (valsartan/sacubitril) and sinoatrial node modulators (ivabradine) [47]. Other promising upstream or downstream signaling targets are under intensive research and may later contribute to a better understanding of disease pathology, and may offer novel therapeutic options as well.

## 4. Materials and Methods

### 4.1. Animal Model

Male CAL-NZW rabbits weighing 2700–3000 g (aged 20 weeks, *n* = 18) were ordered from Jurasko Ltd. (Debrecen, Hungary). Crossing New Zealand White (NZW) with Californian (CAL) rabbits result in increased average body fat content and elevated body weight [48], thus we hypothesized the higher value of this rabbit strain for the current study in imitating the human disorder.

The animals were housed under a 12 h-12 h light-dark cycle and were kept on normal chow until baseline data was recorded. Control rabbits received normal rodent chow and HC animals were kept on “atherogenic” diet. “Atherogenic” chow contained 1% cholesterol and 1% additional saturated fat, formulated in the Dept. of Pharmaceutical Technology at the University of Debrecen. All experimental protocols were approved by the local Ethics Committee of University of Debrecen and the animals received humane care in accordance with the “Principles of Laboratory Animal Care” by EU Directive 2010/63/EU (permission: 25/2013DEMÁB; 29 January 2014). Before starting the experimental protocol, a 2-week adaptation period was provided.

### 4.2. Chemicals

All chemicals, reagents and buffer solutions used for Western blot and cGMP assay were obtained from Sigma-Aldrich-Merck KGaA (Darmstadt, Germany), and Abcam Plc. (Cambridge, UK). For vascular assays, the following chemicals were used: norepinephrine hydrochloride (NE; as Arterenol^®^), acetylcholine chloride (Ach), adenosine 5′-triphosphate disodium salt hydrate (ATP) and chemicals for Krebs solution (purchased from Sigma-Aldrich-Merck KGaA, Darmstadt, Germany). Krebs solution contained (in mmol/L): NaCl: 118, KCl: 4.7, CaCl_2_: 2.5, NaH_2_PO_4_: 1, MgCl_2_: 1.2, NaHCO_3_: 24.9, glucose: 11.5, and ascorbic acid: 0.1, dissolved in redistilled water. Krebs solution was used to dissolve Ach and ATP, and to dilute the NE solution. CaCl_2_-free Krebs buffer was used to wash isolated organs for further studies.

### 4.3. Study Design

Rabbits were randomly divided into 2 groups: “Control” group received normal rabbit chow for 4 months (*n* = 9); “HC” group received “atherogenic” rabbit chow supplemented with 1% cholesterol and 1% fat (*n* = 9). Baseline data of 9 random animals were recorded before the start point (defined as group “Baseline”), After 4 months, endpoint parameters (body weight, echocardiographic data, serum biomarkers) were measured and rabbits were sacrificed by thoracotomy under ketamine-xylazine (50/5 mg/kg) anesthesia. Functional vascular assays were carried out on isolated aortic rings. Morphometric measurements (weighing of organs) were performed, and tissue samples were rapidly frozen in liquid N_2_ and stored at −80 °C for Western blot analyses and cGMP assay (left ventricle), or were fixed in 4% formalin solution for histology (heart, abdominal aorta, kidney, lung and liver samples).

### 4.4. Echocardiographic Studies

Echocardiography was performed under anesthesia (ketamine 35 mg/kg, xylazine 3 mg/kg, i.m.), as described previously [49]. The chest hair was shaved and the rabbits were positioned in a dorsal position without restraint. Data acquisition was performed using a Vivid E9 ultrasound machine (GE Healthcare, New York, NY, USA), with a 12S-D probe suitable for rodent models. The Minimum Transthoracic Echo dataset (2-dimensional, M-mode, Doppler (PW), and tissue Doppler (TVI) recordings from parasternal long- and short- axis, as well as apical 4 chamber views) were acquired as recommended by the American Society of Echocardiography [50]. Additionally, strain analyses were also performed from apical long axis and 4-chamber views (Section 4.5). Recordings were analyzed using EchoPAC PC software (ver. 112, GE Healthcare, New York, NY, USA) by a blinded expert. Weight of the left ventricle was calculated by the equation: LV Mass (g) = 0.8 × (1.04 × [([LVIDd + IVSd + LVPWd]^3^ − LVIDd^3^)]) + 0.6. To assess the geometry of ventricular remodeling (concentric or eccentric), relative wall thickness (RWT) was calculated using the formula: RWT = 2 × (LVPWd)/LVIDd, and increased RWT was considered to show concentric hypertrophy, as it is suggested by the American Society of Echocardiography’s Guideline.

### 4.5. Strain (Speckle Tracking) Analyses

Speckle tracking method was performed offline from apical long axis (APLAX) and 4 chamber views (4CH) by using both EchoPAC Q-analysis/2DStrain measurement option, and the Automatic Functional Imaging (AFI) mode of the software, which is an automated tracking algorithm that follows the myocardial fibers and generates strain and strain rate data. AFI and Q-analysis data were well-correlated. Region of interest (ROI) was manually corrected, peak systolic longitudinal strain (PSLS) was calculated, and the average of values was considered as global longitudinal strain (GLS), that sensitively describes LV systolic function [51].

### 4.6. Analysis of Serum Parameters

Blood samples were collected from the marginal ear veins of animals in Vacutainer Plast SSTII tubes (Becton, Dickinson and Company, Franklin Lakes, NJ, USA), after 12-h fasting. The samples were collected and processed aseptically to minimize hemolytic activity. Insulin was measured on the Liaison XL DiaSorin platform (DiaSorin Inc., Stillwater, MN, USA). All other serum parameters were measured on the Roche Cobas Integrated platform (Roche Diagnostics GmbH, Mannheim, Germany). Lipid parameters included: total cholesterol, low-density lipoprotein (LDLc), high-density lipoprotein (HDLc), apolipoprotein A and B (ApoA and ApoB), and triglyceride. Liver enzymes included: aspartate transaminase (AST) and alanine transaminase (ALT). Specific markers were creatinin, troponin T, creatine kinase MB isoform (CK-MB) and C-reactive protein (CRP). Atherogenic index (total cholesterol/HDLc) and ApoB to ApoA ratios were calculated. Serum NT-pro-BNP was determined by sandwich electrochemiluminescence immunoassay (ECLIA) on the Roche Cobas integrated platform.

### 4.7. Morphometry

At the endpoint of the study, all animals were weighed, anaesthetized deeply with ketamine-xylazine (50/5 mg/kg) combination, and thoracotomy was performed. Organ samples (heart, lung, kidney, liver and right tibia) were excised, washed in Ca^2+^-free Krebs buffer, and immediately weighed using a milligram scale. Left ventricle and septum of the hearts were precisely isolated, weights were measured separately and were normalized to tibial length. Kidney and lung samples were weighed, kept overnight at 60 °C and wet to dry tissue ratios were calculated. Organ samples that remained, as well as the thoracic aorta were cut in half and stored in 4% formalin solution or were rapidly deep-frozen in N_2_ and stored at −80 °C for further analyses.

### 4.8. Functional Vascular Assays

Immediately after sacrificing, the distal part of the thoracic aorta was isolated and 2-mm-wide rings were cut off (four rings from each animal). The rings were mounted horizontally at 10 mN resting tension, using a wire instrument (Experimetria Ltd., Hungary), in a 4-chamber isolated organ bath system with 10 mL vertical chambers (TSZ-04; Experimetria Ltd., Hungary) containing Krebs solution, oxygenated with 95% O_2_ and 5% CO_2_ (36 °C; pH = 7.4). The isometric contractile force of the circulatory muscle layer was measured by a transducer (SD-01; Experimetria Ltd., Hungary) connected with a WS-DA-02 workstation (MDE Research) with SPEL Advances Isosys software (SOFT-02; MDE GmbH, Heidelberg, Germany) [52].

After a 60-min incubation period, a NE concentration-response (E/c) curve (from 1 nmol/L to 10 µmol/L) was generated on the aortic rings to determine their half maximal effective concentration (EC50) value. This was followed by a 60-min wash-out, then EC50 for NE was administered to the rings (pre-contraction). After, Ach E/c curve was constructed (from 0.1 nmol/L to 1 mmol/L). ATP E/c curve (0.1–100 µmol/L) was constructed in the same manner [52].

Responses of vessels obtained from the same animal were averaged. The effect of NE was defined as an increase of the contractile force in addition to the resting tension (10 mN) of aortic rings. If relaxation occurred in the presence of a certain Ach concentration, the maximal relaxation was taken into account. If contraction occurred, its maximum was considered. The effect of Ach and ATP was defined as a percentage change in the initial tension of the aortic ring (after the pre-contraction with NE using its EC50 value related to the given ring).

Ach E/c curves were compared in two manners. First, effect values belonging to the same concentration values were compared between the two experimental groups (NE and ATP E/c curve). Secondly, to quantify the NO-dependent relaxation separated from the NO-independent contraction (developed at higher Ach concentrations), Ach E/c data cleansing was performed as described elsewhere [52]. Briefly, for each individual Ach E/c curve (resulted from averaging the data of the four aortic rings obtained from the same animal), the maximal relaxation effect value was chosen and was arbitrarily assigned to all Ach concentrations that were higher than that belonging to the maximal relaxation effect value (up to 10 µmol/L). Furthermore, to make the regression more reliable, zero effect was assigned to two surely ineffective Ach concentrations (1 and 10 fmol/L), and these E/c data were arbitrarily added to each individual Ach E/c data set. These modified Ach E/c curves were fitted to the Hill equation [53] and the three regression parameters were compared among groups.

### 4.9. Histology

Aorta (root and ascending part), heart and liver samples (fixed in 4% neutral buffered formalin for 24 h) were further processed for histological evaluation as described previously [54]. Briefly, the tissues were embedded in paraffin and sectioned into 7 μm thick slices. Hematoxylin-eosin was used to stain aorta cross-sections (to determine plaque coverage) and liver sections (to show steatosis). Interstitial fibrosis was visualized in heart tissue samples using a Masson’s trichrome stain kit, based on the protocol provided by the manufacturer (Sigma-Aldrich Co., St. Louis, MO, USA).

### 4.10. Western Blot

Western blot was carried out to estimate protein expression levels (PDE9A; glyceraldehyde 3-phosphate dehydrogenase (GAPDH); vasodilator-stimulated phosphoprotein (VASP); phospho-VASP at Ser^239^ (pVASP); and Protein kinase G (PKG)), according to the procedure described elsewhere by the authors [55]. Proteins were isolated from stored left ventricle tissues and from thoracic aorta in buffer solution containing protease inhibitor cocktail. solution (containing 25 mM Tris, 25 mM NaCl, 0.5 mM EDTA, protease inhibitor cocktail X and distilled water), using a homogenizer (IKA-WERKE, Staufen, Germany). Samples were then centrifuged at 10,000× *g* for 20 min. Total protein concentration was measured using an automated spectrophotometer (FLUOstar Optima, BMG Labtech, Ortenberg, Germany). Samples were separated by SDS-polyacrylamide (10, 12 or 18%) gel electrophoresis at 40 mA for 100–120 min, then were transferred to a nitrocellulose membrane (GE Heathcare, New York, NY, USA). The membranes were blocked in 3% BSA blocking buffer and then incubated overnight with primary antibodies (anti-GAPDH, anti-PDE9A, anti-VASP, anti-pVASP, and anti-PKG for myocardial samples; and anti-PDE9A for aortic samples; antibodies obtained from Sigma-Aldrich (Sigma-Aldrich-Merck KGaA, Darmstadt, Germany) and Abcam (Abcam Plc., Cambridge, UK), then incubated with horseradish peroxidase-labeled secondary antibodies (Sigma-Aldrich-Merck KGaA, Darmstadt, Germany). Enhanced chemiluminescent substrate (WesternBright™, ECL, Advansta Inc., Menlo Park, CA, USA) was used to identify bands. Detection was made by a C-Digit® blot scanner with Image Studio Digits ver. 5.2. software (LI-COR Inc., Lincoln, NE, USA).

Quantitative analysis of scanned blots was carried out using the ImageJ software (ver. 1.51k., National Institutes of Health, USA). Signal intensities for the bands were normalized to the background and were standardized to a housekeeping protein (GAPDH). Samples were analyzed three times in independent blots. Quantification was performed, measurements were averaged and Student’s *t*-test was used to estimate differences between signal intensities and corresponding protein expression levels.

### 4.11. cGMP Assay

Quantification of cGMP in LV samples were carried out by a direct competitive immunoassay (Abcam Plc., Cambridge, UK). After preparing standard curve, samples (*n* = 6 per group, each measured in duplicates) were homogenized in 0.1 M HCl. All steps were made as recommended by the manufacturer. The amount of competing cGMP-HRP conjugates bound to the plate was measured by reading optical density (OD) at 450 nm using a spectrophotometer (FLUOstar Optima, BMG Labtech, Ortenberg, Germany). The amount of cGMP was averaged, and expressed as pmol/mg tissue in each group. Student’s *t*-test was used to estimate differences between Control and HC animals.

### 4.12. Statistical Analyses

All data are presented as the average outcome in a group (mean) ± standard error of the mean (SEM). The D’Agostino-Pearson normality test was used to estimate Gaussian distribution, and statistical analysis was then performed using unpaired Student’s *t*-test or Mann-Whitney test (when normality test was not passed). Analyses were carried out using GraphPad Prism software for Windows, version 6.01 (GraphPad Software Inc., La Jolla, CA, USA). Probability values (*p*) less than 0.05 were considered significantly different, and asterisks show the level of significance compared to Controls (* *p* < 0.05; ** *p* < 0.01; *** *p* < 0.001; and **** *p* < 0.0001).

## 5. Conclusions

Here, we generated a rabbit model maintained on atherogenic diet, that shows signs of cardiac dysfunction, including LV relaxation abnormalities, increased afterload, interstitial fibrosis, concentric hypertrophy and endothelial dysfunction. The primary outcome of this study is that upregulation of myocardial and vascular phosphodiesterase 9A isoform (PDE9A), along with cardiac upregulation of vasodilator-stimulated phosphoprotein (VASP) and Protein Kinase G (PKG1) under atherosclerotic conditions. As myocardial cGMP content and relative phosphorylation of VASP protein did not decrease despite of the upregulated PDE9A and PKG1 in this model, further investigations are needed to evaluate the role of phosphodiesterases and the cGMP-PKG system in the pathology of cardiac dysfunction. Questions raised here may be answered by evaluating the effects of PDE9A inhibitors in atherosclerotic cardiovascular disease, which may outline future directions to better understand pathological changes and may contribute to expand opportunities in drug development in the field of atherosclerosis-related disorders.

## Figures and Tables

**Table 1:** Echocardiographic (2D, M-mode and LVOT) parameters of animals at the endpoint of the study. Significant differences were found in LA area, LV mass, RWT, as well in LVOT pressures and velocities in HC animals compared to Control. Asterisks denote the level of significance, compared to the Control (* *p* < 0.05; ** *p* < 0.01; *** *p* < 0.001; **** *p* < 0.0001). LV: left ventricle; LA: left atrium; Ao: aortic; RWT: relative wall thickness; LVFS: left ventricle fractional shortening; LVEF: left ventricle ejection fraction; MAPSE: mitral annular plane systolic excursion; LVOT: left ventricle outflow tract; Vmax: maximal velocity, Vmean: mean velocity; maxPG: maximal pressure gradient; meanPG: mean pressure gradient; HR: heart rate.

**2D and M-Mode**	**Baseline (*n* = 9) Start**	**Control (*n* = 9) Endpoint**	**HC (*n* = 9) Endpoint**	***p* Value (HC vs. Control)**
LA area (cm^2^)	0.789 ± 0.054	0.833 ± 0.029	1.633 ± 0.065 ****	<0.0001
IVSd (mm)	2.153 ± 0.074	2.27 ± 0.139	2.882 ± 0.176 *	0.0149
LV mass (g)	4.057 ± 0.292	4.651 ± 0.433	8.035 ± 0.655 ***	0.0005
RWT (%)	0.260 ± 0.020	0.283 ± 0.017	0.362 ± 0.018 **	0.0050
LV FS (%)	32.1 ± 0.706	30.78 ± 0.434	28.44 ± 0.669 **	0.0099
LV EF (%)	63.9 ± 0.994	62.22 ± 0.619	58.33 ± 0.898 **	0.0026
MAPSE (mm)	5.263 ± 0.209	5.488 ± 0.093	5.661 ± 0.254	0.5147
**LVOT Parameters**	**Baseline**	**Control**	**HC**	***p* Value**
Vmax (m/s)	0.884 ± 0.016	0.923 ± 0.018	1.204 ± 0.021 ****	<0.0001
Vmean (m/s)	0.542 ± 0.012	0.601 ± 0.018	0.701 ± 0.027 **	0.0076
maxPG (mmHg)	3.141 ± 0.117	3.453 ± 0.139	5.832 ± 0.199 ****	<0.0001
meanPG (mmHg)	1.478 ± 0.054	1.747 ± 0.104	2.637 ± 0.172 ***	0.0004

**Table 2:** Echocardiographic (Doppler, TVI, and speckle tracking) parameters of rabbits at the endpoint of the study. Significant differences were found in E/A and E/e’ ratios, DecT, IVRT, Tei-index and GLS in HC animals compared to Controls. Asterisks denote the level of significance, compared to the Control (** *p* < 0.01; *** *p* < 0.001; **** *p* < 0.0001). MV: mitral valve; ms: milliseconds; E vel: peak velocity flow in early diastole A vel: peak velocity flow in atrial contraction (late diastole); E/A ratio: ratio of peak velocity flow in early diastole to peak velocity flow in atrial contraction; DecT: deceleration time of the E wave, Sept: septal; e’: peak velocity of early diastolic annular motion; a’: peak velocity of diastolic annular motion; e’/a’: ratio of e’ to a’; E/e’ ratio: ratio of E to e’; IVRT: isovolumic relaxation time; MPI: myocardial performance index; GLS: global longitudinal strain.

**Doppler and TVI**	**Baseline (*n* = 9) Start**	**Control (*n* = 9) Endpoint**	**HC (*n* = 9) Endpoint**	***p* Value (HC vs. Control)**
MV E vel (m/s)	0.707 ± 0.036	0.630 ± 0.022	0.698 ± 0.047	0.2049
MV A vel (m/s)	0.399 ± 0.023	0.391 ± 0.012	0.653 ± 0.042 ****	<0.0001
E/A ratio	1.836 ± 0.139	1.624 ± 0.068	1.069 ± 0.039 ****	<0.0001
DecT (ms)	58.30 ± 2.864	55.44 ± 2.001	71.22 ± 2.666 ***	0.0002
Sept e’/a’ ratio	1.538 ± 0.078	1.233 ± 0.060	0.935 ± 0.054 **	0.0020
E/e’ ratio	8.311 ± 0.165	8.002 ± 0.164	13.20 ± 1.091 ***	0.0002
IVRT (ms)	34.10 ± 1.690	30.78 ± 1.188	56.56 ± 2.588 ****	<0.0001
Tei-index (MPI)	0.560 ± 0.017	0.528 ± 0.013	0.8385 ± 0.049 ****	<0.0001
**Strain Data**	**Baseline**	**Control**	**HC**	***p* Value**
GLS	−22.50 ± 0.806	−21.34 ± 0.664	−16.07 ± 1.029 ***	0.0007

**Table 3:** Body-, heart- and left ventricle weight, LV mass to tibial length ratios, lung and kidney wet to dry tissue ratios at the endpoint of the study. Asterisks denote the level of statistical significance compared to Control (** *p* < 0.01; *** *p* < 0.001; Student’s *t*-test).

Parameter	Control (*n* = 9)	HC (*n* = 9) Endpoint
Bodyweight (g)	3064 ± 87	4153 ± 86 ***
Heart weight	7.795 ± 0.418	12.25 ± 0.535 ***
Heart weight to BW ratio	0.00253 ± 0.0003	0.00294 ± 0.0002
Left ventricle mass (g)	6.22 ± 0.337	10.13 ± 0.534 ***
LV mass to tibial length	0.605 ± 0.046	0.828 ± 0.038 **
LV mass to BW ratio	0.00087 ± 0.0003	0.0008 ± 0.0002
Lung wet to dry ratio	5.254 ± 0.147	5.118 ± 0.094
Kidney wet to dry ratio	3.421 ± 0.058	3.281 ± 0.191

**Table 4:** Serum parameters. Serum lipid parameters increased dramatically in the HC group, as well as atherogenic index and ApoB/ApoA ratio. Troponin T levels were elevated, but did not reach the level referring to acute myocardial infarction. Student’s *t*-test was used to estimate differences between endpoint groups. Asterisks denote the level of statistical significance compared to Control (* *p* < 0.05; ** *p* < 0.01; *** *p* < 0.001; **** *p* < 0.0001). Total chol.: total cholesterol, LDLc: low-density lipoprotein; HDLc: high-density lipoprotein, ApoA: apolipoprotein A; ApoB: apolipoprotein B; AST: aspartate transaminase; ALT: alanine transaminase; CK-MB: creatine kinase MB isoform; CRP: C-reactive protein. Atherogenic index was defined as total cholesterol/HDLc.

Serum Parameter	Baseline (*n* = 9) Start	Control (*n* = 9) Endpoint	HC (*n* = 9) Endpoint	*p* Value (HC vs. Control)
Total chol. (mmol/L)	3.440 ± 0.199	1.753 ± 0.0187	32.360 ± 2.164 ****	<0.0001
LDLc (mmol/L)	2.759 ± 0.317	0.725 ± 0.312	32.030 ± 1.813 ****	<0.0001
ApoB (mmol/L)	0.057 ± 0.008	0.026 ± 0.005	0.154 ± 0.006 ****	<0.0001
HDLc (mmol/L)	1.188 ± 0.212	0.963 ± 0.092	6.428 ± 0.600 ****	<0.0001
ApoA (mmol/L)	0.112 ± 0.024	0.149 ± 0.011	0.034 ± 0.007 ****	<0.0001
Atherogenic index	2.457 ± 0.170	1.986 ± 0.391	5.499 ± 0.458 ***	0.0001
ApoB to ApoA ratio	0.385 ± 0.046	0.188 ± 0.041	3.691 ± 0.492 ****	<0.0001
Triglyceride (mmol/L)	0.410 ± 0.066	1.139 ± 0.136	1.001 ± 0.225	0.6959
AST (GOT) (U/L)	25.000 ± 5.177	22.710 ± 6.781	33.47 ± 3.016	0.1056
ALT (GPT) (U/L)	50.830 ± 8.284	64.140 ± 19.660	57.730 ± 5.479	0.6813
Creatinine (μmol/L)	83.000 ± 6.033	71.430 ± 12.040	116.100 ± 5.984 **	0.0013
CK-MB (U/L)	1077.0 ± 384.7	1154.0 ± 276.9	785.6 ± 126.1	0.1754
Insulin (µIU/mL)	17.63 ± 0.41	23.70 ± 2.46	49.38 ± 6.70 **	0.0089
Glucose (mmol/L)	4.917 ± 0.202	7.100 ± 0.245	6.918 ± 0.278	0.7178
CRP (mg/L)	0.251 ± 0.075	0.079 ± 0.070	0.325 ± 0.073 *	0.0359
Troponin T (ng/L)	5.528 ± 1.010	7.438 ± 1.199	20.21 ± 2.778 **	0.0092

**Table 5:** Regression parameters (expressed in mean ± SEM) of the Hill equation fitted to the individual modified acetylcholine (Ach) concentration-response (E/c) curves. Level of statistical significance for differences between the Control and HC groups is indicated (* *p* < 0.05; ** *p* < 0.01). *E*_max_: maximal effect; logEC_50_: common logarithm of EC_50_, the concentration producing half-maximal effect; n: Hill coefficient (slope factor).

Parameter	Control	HC
**Emax**	93.67 ± 2.66	50.9 ± 9.48 **
**logEC50**	−8.62 ± 0.36	−7.59 ± 0.16 *
**n**	0.91 ± 0.19	1 ± 0.11
